# Characterization of potential spermatogonia biomarker genes in the European eel (*Anguilla anguilla*)

**DOI:** 10.1007/s10695-024-01338-1

**Published:** 2024-04-19

**Authors:** Marta Blanes-García, Zoran Marinović, Juan Germán Herranz-Jusdado, Xuan Xie, Leonor Ferrão, Victor Gallego, Luz Pérez, Abdul Rasheed Baloch, Ákos Horváth, Martin Pšenička, Juan F. Asturiano, Marina Morini

**Affiliations:** 1 Grupo de Acuicultura y Biodiversidad, Instituto de Ciencia y Tecnología Animal, Universitat Politècnica de València, Camino de Vera S/N, 46022 Valencia, Spain; 2 Department of Aquaculture, Institute of Aquaculture and Environmental Safety, Hungarian University of Agriculture and Life Sciences, Páter Károly U. 1, 2100 Gödöllő, Hungary; 3 Faculty of Fisheries and Protection of Waters, South Bohemian Research Center of Aquaculture and Biodiversity of Hydrocenoses, University of South Bohemia in Ceske Budejovice, Zátiší 728/II, 389 25 Vodňany, Czech Republic

**Keywords:** Testis, Fish, Gene expression, *Vasa*, *Dnd1*, *Nanos2*

## Abstract

Identification of specific molecular markers for spermatogonial stem cells in teleost is crucial for enhancing the efficacy of reproductive biotechnologies in aquaculture, such as transplantation and surrogate production in fishes. Since it is not yet possible to distinguish spermatogonial stem cells of European eel (*Anguilla anguilla*) using specific molecular markers, we isolated spermatogonial cells from immature European eels to find these potential markers. We attempted this by studying three candidate genes: *vasa*, *nanos2*, and *dnd1*. Two *vasa* (*vasa1* and *vasa2*) genes, *nanos2*, and *dnd1* were identified, characterized, and studied in the muscle, testis, and isolated spermatogonia. Our results showed that *vasa1* and *vasa2* had the highest levels of expression when measured by qPCR. In situ hybridization and immunochemistry assays showed that the four genes were localized explicitly in type A spermatogonia. However, *vasa1* and *vasa2* exhibited stronger signals in the immature testicular tissue than the other two potential markers. According to this, *vasa1* and *vasa2* were found to be the most effective markers for spermatogonial cells in the European eel.

**Supplementary Information:**

The online version contains supplementary material available at 10.1007/s10695-024-01338-1.

## Introduction

The European eel (*Anguilla anguilla*) has a complex catadromous life cycle that includes a long reproductive migration across the Atlantic Ocean to reach their spawning site in the unknown areas of the Sargasso Sea (Schmidt 1923). During the last decades, different factors have significantly impacted eel populations, resulting in a decline in the continental stocks of the eels (Dekker 2002). As a result, the European eel was included in 2007 in the Appendix II of CITES and the Red list of IUCN. Currently, eel production in aquaculture is based on extractive fishing, as their production cycle has not yet been closed. Therefore, to reduce pressure on eel stocks, reproduction in captivity is required. One of the main issues for the reproduction in captivity of the eels is that the reproductive development is blocked in the pre-pubertal stage until their oceanic reproductive migration starts (Dufour et al. 1988). In general, limited development of gonads has been recorded when eels first migrate to oceans (gonadosomatic index = 1–2), although there are slight differences depending on the environmental conditions (Gentile et al. 2022; Palstra et al. 2011). Keeping these eels in captivity does not enhance further gonadal development, indicating the essentiality of environmental triggers, such as photoperiod, salinity, and temperature regimes, for sexual maturation (Burgerhout et al. 2019; van Ginneken and Maes 2005). Achieving sexual maturation in captive conditions requires long hormonal treatments, limited by a great variation in the response of each eel (reviewed by Asturiano 2020). Regarding this, it is crucial to understand the molecular mechanisms involved in germ cell development to make advances in the reproduction of the European eel. For instance, spermatozoa production depends on the subpopulation of spermatogonial stem cells, which in fishes are considered to be undifferentiated type A spermatogonia (Schulz et al. 2010). Studying molecular markers for spermatogonial stem cells in fish provides a potential application for new reproductive biotechnologies, such as type A spermatogonia (SPGA) transplantation, in vitro spermatogenesis or reprogramming of spermatogonial cell lines into pluripotent cells (Robles et al. 2017; Thoma et al. 2011). Several proteins have been identified in spermatogonial populations in certain fish species (Bosseboeuf et al. 2014; Lacerda et al. 2013; Nagasawa et al. 2012; Ozaki et al. 2011). Some candidates, such as Notch1 (Yano et al. 2009), Ly75 (Nagasawa et al. 2010), Plzf (Ozaki et al. 2011), Oct-4 (Sánchez-Sánchez et al. 2010), and SGSA-1 (Kobayashi et al. 1998), have been proposed, but specific molecular markers for fish spermatogonial stem cells have yet to be discovered (Lacerda et al. 2018).

Vasa, or DDX4 (DEAD-box helicase 4), belongs to the RNA helicase of the DEAD (Asp-Glu-Ala-Asp) family. Vasa is implicated in the translational control of RNA activation (Hay et al. 1988; Lasko and Ashburner 1988). It has been identified in various metazoan species, such as cnidaria (Mochizuki et al. 2001), non-vertebrate Ecdysozoa (Schüpbach and Wieschaus 1986), mammals (Castrillon et al. 2000), or teleost species (Cao et al. 2012), and has been reported to be a germ cell marker in the animal kingdom.

Dead end (Dnd) protein encodes an RNA-binding protein (RBP) essential for primordial germ cell (PGC) migration and gametogenesis in vertebrates. In 2003, *dnd1* was recognized as a component of zebrafish (*Danio rerio*) germplasm (also called germ granules or nuage) that is specifically expressed in PGCs throughout embryogenesis (Weidinger et al. 2003). The Dnd1 protein is localized in perinuclear germ granules within PGCs, helping their polarization, motility, and survival.

*Nanos* is one of the essential genes for the development of germ cells. *Nanos2* expression was found to be restricted to the gonads of adult mammalian males and has been reported to be a specific marker of the male germinal stem cells of mice. In medaka (*Oryzias latipes*) and zebrafish, ovarian germ stem cells and spermatogonia in adult gonads exhibited expression of *nanos2* (Aoki et al. 2009; Draper 2017; Nakamura et al. 2010).

In our present study, we aimed to determine spermatogonia molecular markers by identifying *vasa*, *nanos2*, and *dnd*1 genes in the testicular tissue of immature European eels. In addition, we studied the expression pattern of these genes in the testis, in isolated spermatogonia, and muscles, and have determined their distribution within the testicular tissue through fluorescent in situ hybridization (FISH), immunohistochemistry, and immunocytochemistry.

## Material and methods

### Fish handling and sampling

Farmed immature European eels were bought alive at a local supermarket and transported to the facilities in the Laboratory of Fish Reproduction of the Universitat Politècnica de València (Valencia, East coast of Spain). Fish were anaesthetized upon arrival with benzocaine (60 ppm) and euthanized by decapitation. The maturation status of the eels was visually verified by checking the shading of the pectoral fin, which was transparent because of the immature stage of the eels (Okamura et al. 2007; Peñaranda et al. 2018). After dissection, only males were selected. Testes (*n* = 39) and muscle (*n* = 23) tissue samples were collected and stored in RNAlater (Ambion-Inc., Austin, TX, USA) and kept at − 20 °C until RNA extraction.

### Isolation of spermatogonia

Testes from immature male European eels (*n* = 13) were dissected and washed with a phosphate-buffered saline (PBS), and the abdominal fat was removed. Testes were cut into small pieces and then transferred into 15-mL tubes containing 10 mL PBS and 0.2% trypsin for enzymatic dissociation. The mixture was incubated for 2 h at 22 °C. DNA was eliminated by adding 40 µg/mL DNase I (Pan-Reac AppliChem, Spain). Between 10 and 12 pipetting of the mixture was performed every 30 min to get better tissue dissociation. The obtained suspension was then filtered with a 40-µm meshed filter to separate debris, and the enzymatic reaction was stopped by adding 1% bovine serum albumin (BSA; Sigma-Aldrich).

To enrich the percentage of spermatogonial stem cells, a Percoll density gradient was employed. The Percoll solution at 33% (v/v) was prepared and transferred into a 50-mL plastic tube. The cell suspension was slowly transferred to tubes avoiding mixing layers. After that, the suspension was centrifuged at 400 × g for 20 min at 20 °C using a progressive acceleration and deceleration centrifuge program. When completed, the middle layer containing spermatogonia was extracted from the mixture and transferred to a 15-mL plastic tube. It was then diluted 3 × in PBS and centrifuged again at 400 × g, 20 °C for 20 min. After this second centrifugation, the supernatant was carefully removed, and the pellet containing the testicular cells was resuspended in PBS. The obtained testicular cells were observed under a light optical microscope, and due to their spherical appearance, large size (10–20 µm), and large nuclei (6–10 µm) (Robles et al. 2017), the germ cells at the early developmental stage were distinguished from other cell types, and their number was counted using a Neubauer cell hemocytometer at × 40 magnification. Cell viability was evaluated by adding 0.4% trypan blue.

### RNA extraction and reverse transcription

RNA from the enriched spermatogonia fraction was extracted immediately after their isolation, while testis and muscle RNA were extracted from samples kept in RNAlater. Total RNA was isolated using phenol/chloroform extraction in the Trizol reagent (Life Technologies, Inc., Carlsbad, CA) according to Peñaranda et al. (2010). RNA concentration and 280/260 and 280/230 ratios were determined using NanoDrop 2000C Spectrophotometer (Fisher Scientific SL, Spain). DNase I treatment and first-strand complementary DNA (cDNA) synthesis were performed from 500 ng of total RNA using a QuantiTect Reverse Transcription kit (Qiagen, Hilden, Germany) following the manufacturer’s instructions.

### Identification of vasa1, vasa2, nanos2, and dnd1 sequences from European and Japanese eel genome datasets

The *vasa1*, *vasa2*, *nanos2*, and *dnd1* sequences in vertebrate species were retrieved from NCBI (http://www.ncbi.nlm.nih.gov/) and Ensembl (https://www.ensembl.org/index.html) databases. All the genomic sequences of *vasa1*, *vasa2*, *nanos2*, and *dnd1* were retrieved from European (*Anguilla anguilla*; for accession/ID number GCF_013347855.1) (Henkel et al. 2012a) and Japanese eel (*Anguilla japonica*) genomes by performing the TBLASTN algorithm of the CLC DNA Workbench software (CLC bio, Aarhus, Denmark) (Henkel et al. 2012b). European eel *vasa1*, *vasa2*, *nanos2*, and *dnd1* sequences were then used to retrieve the genomic sequence coding for the respective genes in the Japanese eel genome (Henkel et al. 2012b). The exons and splicing junctions were predicted using the empirical nucleotidic splicing signatures, that is, intron begins with “GT” and ends with “AG.” The percentage of European and Japanese eel Vasa (DEAD-box helicase 4) and DDX3X (DEAD-box helicase 3X-linked) identity was calculated with the Sequences Identities And Similarities (SIAS) server (imed.med.ucm.es/Tools/sias.html).

### Phylogenetic analysis of vasa1, vasa2, nanos2, and dnd1

Phylogenetic analyses were conducted to retrace the evolutionary scenarios of *vasa*, *nanos2*, and *dnd1* families, as well as to determine and classify the number of paralogs, gene duplications, and gene losses on osteichthyans of key-phylogenetical positions: a representative of an early sarcopterygian, the coelacanth (*Latimeria chalumnae*); mammalians; sauropsids (squamates, chelonians, and archosaurians); the non-teleost actinopterygian spotted gar; and the European and Japanese eels, as members of an early group of teleosts (elopomorphs), and other teleosts. Three phylogenetic trees were constructed with amino acid sequences of known or predicted sequences of Vasa and its closest paralogous gene called DDX3X, Nanos2 (nanos C2HC-type zinc finger 2), and Dnd1 (dead end 1) (for accession/ID number, see Supplementary Table A).

The sequences were retrieved from NCBI or ENSEMBL, first aligned using Clustal Omega (Sievers et al. 2011) with Seaview 4.5.4 software (http://doua.prabi.fr/software/seaview), and later manually adjusted. The JTT (Jones, Taylor, and Thornton) protein substitution matrix of the resulting alignment was determined using ProTest software (Abascal et al. 2005). The alignment of peptide sequences was performed with amino acid sequences of Vasa retrieved from NCBI for European eel (accession numbers XP_035234983.1 and XP_35248267.1), Japanese eel (accession numbers ASV71763.1 and BAO21641.1), and rice field eel (*Monopterus albus*, accession number ABA54551*.*1) to provide further details of *vasa* genes in eels (for alignment, see Supplementary Fig. A).

The three phylogenetic trees were constructed based on the sequence alignments using the maximum likelihood method (PhyML software, (Stamatakis and Ott 2008)) with 1000 bootstrap replicates and subsequently visualized using treedyn (http://phylogeny.lirmm.fr/phylo_cgi/). Human and fugu (*Takifugu rubripes*) DDX10 (DEAD-box helicase 10), human RBM47 (RNA-binding motif protein 47), and human NANOS1 (nanos C2HC-type zinc finger 1) were used as out-group for the Vasa, Dnd1, and Nanos2 phylogenetic analyses, respectively (for accession/ID number, see Supplementary Table A).

### Gene expression analysis by quantitative real-time PCR

Quantitative real-time polymerase chain reactions (qPCR) were carried out using specific qPCR primers for European eel *vasa1*, *vasa2*, *nanos2*, and *dnd1* (Table 1). Acidic ribosomal phosphoprotein P0 (*arp*) was used as a reference gene as previously used for eel (Morini et al. 2015).

**Table 1:** Specific primers sets used for qPCR

Name of gene	5′-3′	Efficiency	Length
*vasa1*	F TTTGGAGGGAGAGGTAGAGG R CTCATTTCCTGATGCGTTCC	105	69
*vasa2*	F GTGTATGAGGTCACCCAGTA R CTCTTGGTCTCTACAAACAC	98	98
*dnd1*	F CGGGACATCTACGAGGACAA R TTCATCATCAGGCGGAACTC	106	77
*nanos2*	F GAGCCAGCAGAGCAGAAA R CCGTCCTTCGCCTTCA	97	194
*arp*	F GTGCCAGCTCAGAACACTG R ACATCGCTCAAGACTTCAATGG	105	107

#### Primers

European eel *vasa1*, *vasa2*, *nanos2*, and *dnd1*–specific qPCR primers were designed based on European eel coding sequences using Primer3 Software (Whitehead Institute/Massachusetts Institute of Technology, Boston, MA, USA). All primers were designed on two different exons to avoid amplifying potential genomic contamination. Primers were purchased from Integrate DNA Technology Inc. (IDT, Coralville, IA).

#### SYBR Green assay

Expression of *vasa1*, *vasa2*, *nanos2*, and *dnd1* genes in spermatogonia, gonads, and muscles (negative control) of immature European eels was measured by performing qPCR assays using a model 7500 unit (Applied Biosystems; Foster City, CA, USA) with Maxima SYBR Green/ROX qPCR Master Mix (Fermentas Corp. Glen Burnie, MD, USA). qPCR program was performed as an initial step of 95 °C for 10 min and 40 cycles of 95 °C for 15 s, 60 °C for 30 s, and 95 °C for 15 s. To evaluate assay specificity, the machine performed a melting curve analysis directly after PCR by slowly (0.3 °C/s) increasing the temperature from 60 to 95 °C, with continuous registration of any changes in fluorescent emission intensity. The total volume for each qPCR reaction was 20 µL, with 5 µL of diluted cDNA (1:20) template, forward and reverse primers (250 nM each), and SYBR Green/ROX Master Mix (12 µL). Serial dilutions of the cDNA pool of gonad tissues were run in duplicate and used for standard curve to measure *vasa1*, *vasa2*, *nanos2*, and *dnd1* in the spermatogonia, testis, and muscle. As a calibrator, a 1/32 standard curve dilution was included in each run of the corresponding gene. Target and reference genes in samples were run in duplicate PCR reactions. A non-template control (cDNA replaced by water) for each primer pair was replicated in all plates. Data for all genes were normalized to eel reference gene *arp*. qPCR calculations were performed according to Roche Applied Science protocol, Technical Note No. LC 13/2001, part 4 “Calibrator normalized relative quantification.”

### Localization analyses

#### Fluorescent in situ hybridization (FISH)

To detect the distribution of the potential spermatogonia markers (*vasa1*, *vasa2*, *nanos2*, and *dnd1*), testes from four immature European eels were dissected. For FISH, RNAscope Multiplex Fluorescent Reagent Kit v2 (Advanced Cell Diagnostics Inc. [ACD], CA, USA) was used following the manufacturer instructions. ACD created probes as *vasa1* (Gene ID 118213421), *vasa2* (Gene ID 118206405), *nanos2* (Gene ID manually characterized using CLC software; see the “RNA extraction and reverse transcription” section), and *dnd1* (Gene ID 118224086) of European eel. *ß-actin* probe, targeting LOC118216518 of European eel ß-actin-1 (Gene ID DQ493907.1), was used as the positive control. Probe diluent was used as the negative control. Testes samples were fixed in 10% neutral buffered formalin for 24 h at room temperature (RT). Then, samples were dehydrated in an increasing percentage of ethanol series (70, 80, 90, 95, 100%) followed by xylene and embedded in resin (Technovit 7100) according to the manufacturer’s instructions. Sections of 5 µm thickness were cut with a Microtome HM325 and were mounted on Superfrost® plus slides (Thermo Fisher Scientific, MA, USA). Two slides were stained with hematoxylin–eosin (H&E) to determine the testis maturation stage (Peñaranda et al. 2010).

After dewaxing and rehydration, antigen retrieval was done by boiling the tissue sections in RNAscope® Target Retrieval Reagents at 98–102 °C for 15 min. Tissue sections were incubated with the appropriate probe at 40 °C for 2 h for probe hybridization. Localization of *vasa1*, *vasa2*, *nanos2*, and *dnd1* RNA transcripts was done by labelling them with Opal 520 fluorophore (Akota Biosciences, MA, USA) by incubating the slides at 40 °C for 30 min. Preliminary testing of optimal fluorophore concentration showed that the 1:750 dilution of the Opal fluorophore displayed a more intense signal than the 1:1000 dilution. Therefore, 1:750 was used in further analyses (see Supplementary Fig. B). Slides were counterstained with DAPI at RT for 30 s, mounted with ProLong Gold Antifade Mountant (Thermo Fisher Scientific, MA, USA) and covered with a coverslip. The slides were stored in the dark at 4 °C. Between 5 and 15 photographs were taken after at least 8 h and until the 2 following weeks using a camera (Moticam 1080, MoticEurope S.L.U, Barcelona, Spain) mounted on a phase-contrast microscope (Nikon Eclipse 80i). Positive signal was visible as fluorescent dots within a cell, correlated with the number of RNA copies. A quantitative analysis according to the manufacturer’s instruction was applied to evaluate results using the image-based software QuPath version 0.4.1 (Bankhead et al. 2017). The software estimated the number of spots per cell counting the number of single spots and clusters. An approximate number of 1500 cells were counted in each photograph.

More detailed photographs were taken using an AxioObserver 780 confocal microscope (Zeiss, Jena, Germany) to diagnose the cell type in which the signal was present.

#### Immunohistochemistry

Three immature European eel males were sacrificed as described above, and their testes were dissected and fixed in modified Davidson’s fixative [mDF; 30% of 37–40% formaldehyde, 15% EtOH, 5% glacial acetic acid in ddH_2_O; (Howroyd et al. 2005)] for 24 h at RT. Tissues were subsequently dehydrated in increasing EtOH series, cleared in xylol and embedded into paraffin. Sections of 5 µm thickness were cut and mounted on Superfrost Ultra Plus® slides (Thermo Fisher Scientific, Massachusetts, USA). One slide was first stained with standard H&E staining to verify the sample’s testicular morphology and identify spermatogonia.

Immunohistochemistry localization of Vasa, Nanos2, and Dnd proteins was done using the 3,3′-diaminobenzidine (DAB) immunoperoxidase visualization method. After dewaxing and rehydration, antigen retrieval was done by boiling the sections in the HistoVT One antigen retrieval solution (Nacalai Tesque Inc, Kyoto, Japan) in a TintoRetriever pressure cooker (Bio SB, Santa Barbara, CA, USA) under the 80–86 ℃ low-pressure setting for 20 min. Endogenous peroxidases were inhibited by treating the sections with 3% H_2_O_2_ in PBS for 30 min, after which non-specific binding was blocked with a blocking solution composed of 10% FBS and 10% goat serum in PBS for 1 h at RT. After the preliminary testing of optimal antibody concentrations, sections were labelled with anti-vasa (1:200; Abcam, ab13840), anti-nanos2 (1:10; Abcam, ab76568), or anti-dnd1 (1:10; Sigma-Aldrich, AV41198) antibodies diluted in the blocking solution, for 1 h at RT. Subsequently, the sections were labelled with a goat anti-rabbit (1:250; Abcam, ab6721) antibody conjugated to horseradish peroxidase for 30 min at RT. The signal was then visualized by applying a solution of 0.05% DAB and 0.015% H_2_O_2_ for approximately 5 min. After washing, the slides were counterstained with hematoxylin for 3 min, dehydrated in increasing EtOH series, cleared in xylol, and mounted with DPX. Sections labelled only with the secondary antibody were considered secondary-antibody control sections, while sections that were not labelled with antibodies but stained with DAB were termed DAB controls. Samples were visualized under the Nikon 6100 Epifluorescent microscope.

#### Immunocytochemistry

Spermatogonia were isolated as described above (see the “Isolation of spermatogonia” section), and the cells were fixed in 10% NBF for 15 min at RT. The cells were centrifuged at 300 × g for 10 min and washed 3 times in PBS. After the last wash, the cells were resuspended in PBS containing 10% polyethylene glycol (PEG) to avoid crystallization of the salts contained in the PBS after evaporation and left overnight at 4 ℃. Approximately 200,000 cells were transferred per well of a chamber slide (Nunc Lab-Tek, NY, USA) and were centrifuged at 1000 × g for 5 min. The supernatant was aspirated, and the cells were left to dry at RT for 2 h. The cells were then permeated by using 0.25% Triton X-100 in PBS for 10 min, and no antigen retrieval was done. As both FISH and immunohistochemistry displayed that *vasa* was more optimal in labelling spermatogonia, cells were labelled only with the anti-vasa (1:200; Abcam, ab13840) antibody for 1 h at RT. Subsequently, the sections were labelled with a goat anti-rabbit (1:500; Abcam, ab150078) antibody conjugated to Alexa 555 for 30 min at RT, counterstained with DAPI, and mounted in 90% glycerol in PBS. Samples were visualized under the Nikon 6100 Epifluorescent microscope.

### Statistics

Results are shown as mean ± standard error of the mean (SEM). Statistical analyses were performed to compare the transcript levels in the muscle, testes, and spermatogonia and the difference in the number of spots estimated between markers. Shapiro–Wilk and Levene tests were used to check the normality of data distribution and variance homogeneity, respectively. Due to the heteroscedasticity of variance, means were compared by the non-parametric Kruskal–Wallis test. Differences were considered significant when *P* < 0.05. All statistical procedures were performed using Statgraphics Plus 5.1 (Statistical Graphics Corp., Rockville, MO, USA).

## Results

### Characterization and phylogenetic analyses of potential spermatogonial biomarkers in eel

#### Vasa and its closest paralog gene DDX3X

The search in European and Japanese eel genomes (Henkel et al. 2012a, b) revealed two eel *vasa* paralogous genes, called *vasa1* and *vasa2*, and two *ddx3x* paralogous genes, called *ddx3x1* and *ddx3x2*, orthologous to the other vertebrate *vasa* and *ddx3x*, respectively. Only partial *vasa2* and *ddx3x2* sequences were achieved from the European and Japanese eel draft genomes. The missing part corresponds to the 3′ start. Two more exons are missing in the middle of the European eel *vasa2* sequence. European eel *vasa1* and *vasa2* display 98.24 and 90% (due to the missing exon in European eel *vasa2*) identity compared to their respective Japanese eel *vasa1* and *vasa2*. European eel *ddx3x1* and *ddx3x2* display 98.31 and 99.33% identity compared to their respective Japanese *ddx3x1* and *ddx3x2*.

A single *vasa* gene has been retrieved in a non-teleost actinopterygian, the spotted gar, as in sarcopterygians and other teleost species. The coelacanth *vasa* is missing in databases. A single *ddx3x* gene has been retrieved in the spotted gar and in the coelacanth, a basal sarcopterygian, as in humans, tetrapods, and some other teleost species. Other teleosts such as fugu (*T. rubripes*), platyfish (*Xiphophorus maculatus*), tilapia (*Oreochromis aureus*), turbot (*Scophthalmus maximus*), salmon (*Salmo salar*), goldfish (*Carassius auratus*), or zebrafish exhibit two *ddx3x* paralogous genes.

**Fig. 1:**
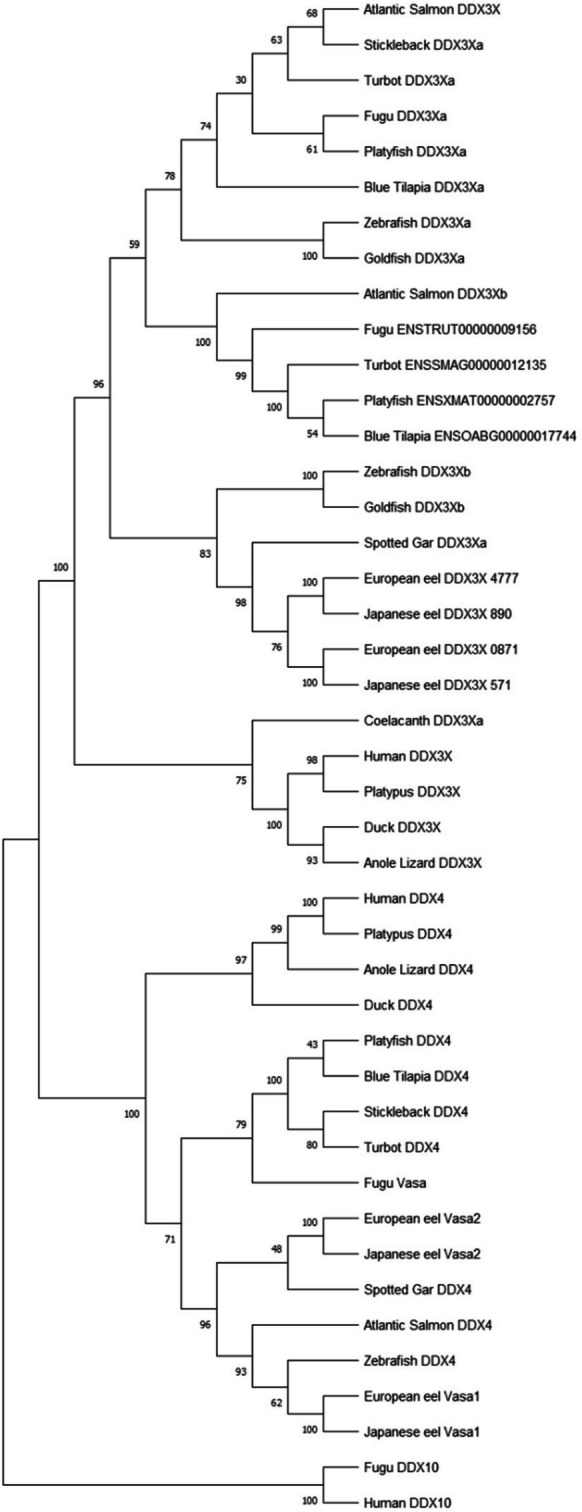
DEAD-box helicase consensus phylogenetic tree. The phylogenetic tree was constructed based on the amino acid sequences of DDX3X (DEAD-box helicase 3X-linked) and Vasa (DEAD-box helicase 4, DDX4) (for the references of each sequence, see Table 1) using the maximum likelihood method with 1000 bootstrap replicates. The number shown at each branch node indicates the bootstrap value (%). The DEAD-box helicase 10 (DDX10) was used as the out-group

In the phylogenetic analyses, Vasa and DDX3X, both members of the DEAD-box helicase family, clustered in two monophyletic groups. In each Vasa and DDX3X monophyletic groups, actinopterygian and sarcopterygian sequences clustered in two well-supported clades (Fig. 1).

#### Nanos2 and dnd1

**Fig. 2:**
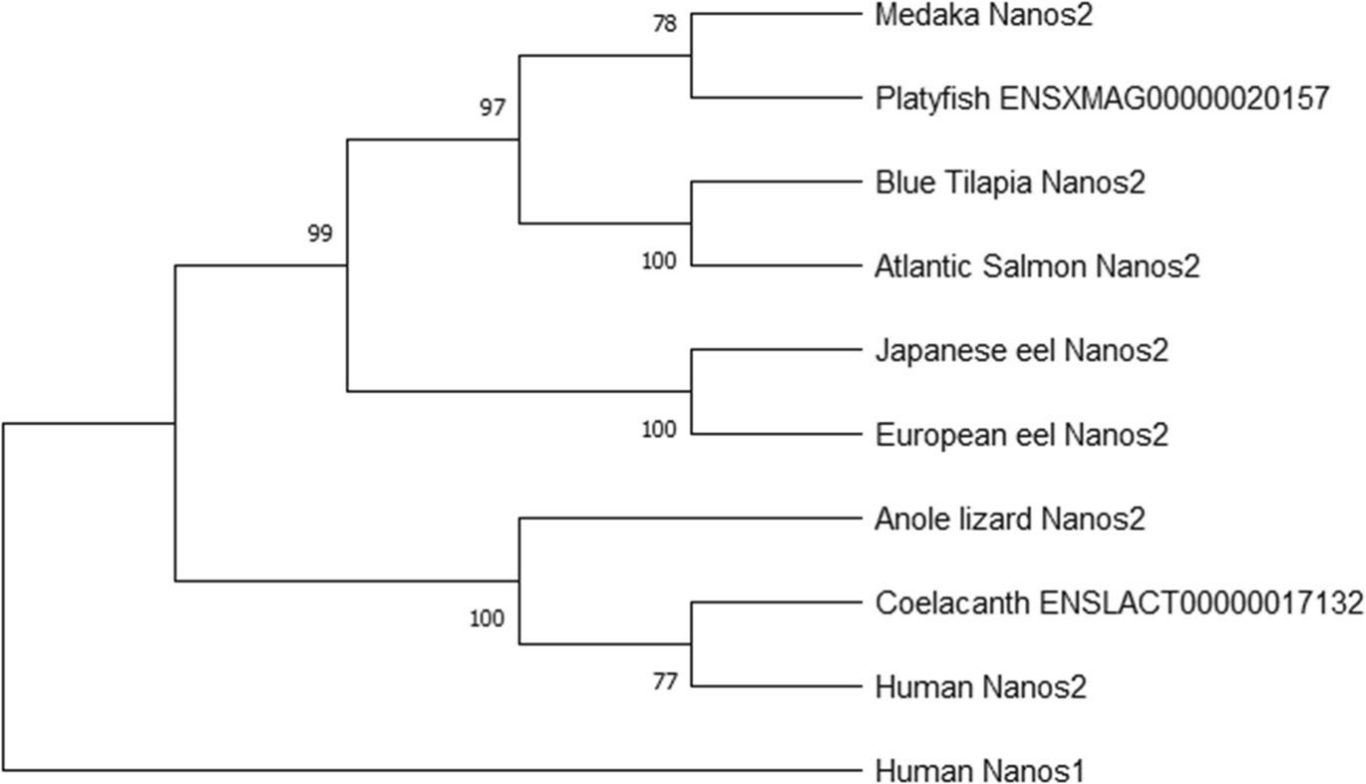
Nanos2 (nanos C2HC-type zinc finger 2) consensus phylogenetic tree. The phylogenetic tree was constructed based on the amino acid sequences of Nanos2 (for the references of each sequence, see Table A) using the maximum likelihood method with 1000 bootstrap replicates. The number shown at each branch node indicates the bootstrap value (%). The Nanos1 (nanos C2HC-type zinc finger 1) was used as the out-group

**Fig. 3:**
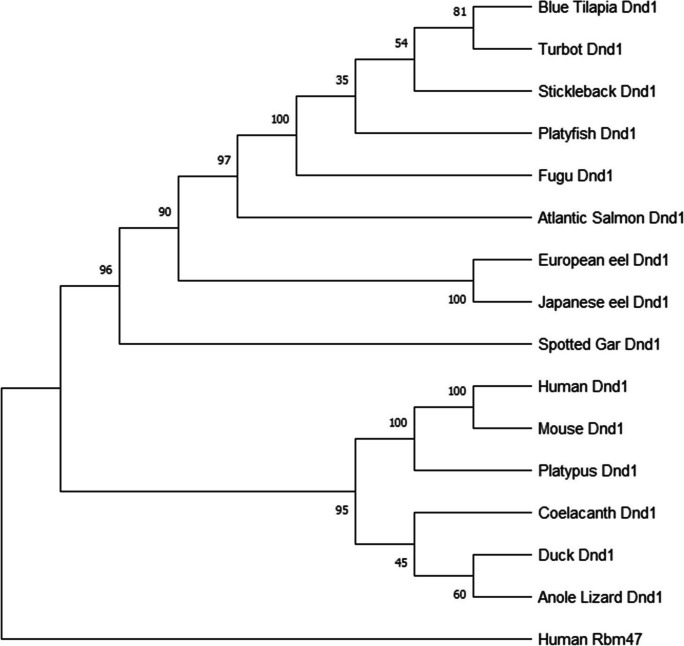
Dnd1 (dead end 1) consensus phylogenetic tree. The phylogenetic tree was constructed based on the amino acid sequences of dnd1 (for the references of each sequence, see Table A) using the maximum likelihood method with 1000 bootstrap replicates. The number shown at each branch node indicates the bootstrap value (%). The RBM47 (RNA binding motif protein 47) was used as the out-group

The search in European and Japanese eel genomes (Henkel et al. 2012a, b) revealed only one eel *nanos2* and one *dnd1* gene, orthologous to the other vertebrate *nanos2* and *dnd1*, respectively. A single *nanos2* and *dnd1* gene has been retrieved in teleost species, as in sarcopterygians. The spotted gar *nanos2* is missing in the database. In both phylogenetic analyses, actinopterygians and sarcopterygians sequences clustered in two monophyletic groups (Figs. 2 and 3).

The two eel Nanos2 sequences form a group branching at the base of the teleost clade, in agreement with the phylogenetical basal position among teleost species (Fig. 2). Similarly, the Dnd1 sequence of the non-teleost actinopterygian, a holostean, the spotted gar, branched at the basis of the actinopterygian clade. Both Japanese and European eel Dnd1 sequences diverged at the base of the other teleost sequences, in agreement with the phylogenetical basal position of elopomorpha among teleosts (Fig. 3).

### Gene expression of potential biomarkers in spermatogonia, testis, and muscle

#### Isolation of spermatogonia

**Fig. 4:**
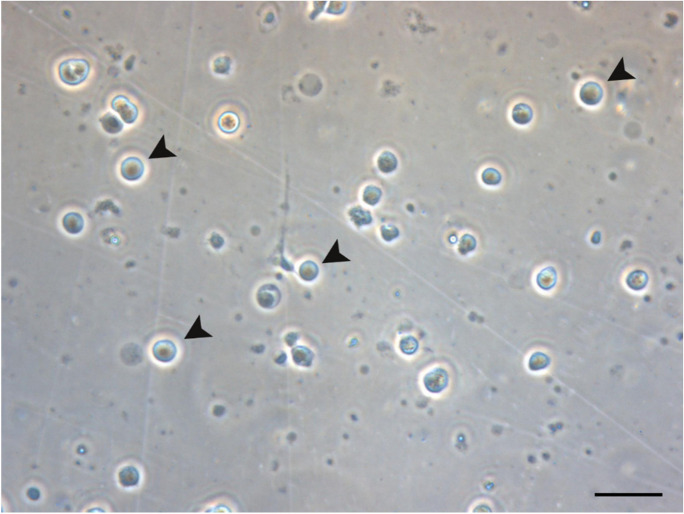
Spermatogonial cells observed under light optical microscope. Spermatogonia are large spherical cells with large nuclei, distinguished from lipids and debris, identified with arrowheads. Scale bar, 25 µm

To measure by qPCR the mRNA expression of 4 different genes, *vasa1*, *vasa2*, *nanos2*, and *dnd1*, in spermatogonia cell population, we previously isolated the spermatogonia cells from the whole testis (Fig. 4). The eels did not receive any treatment that enhances sexual maturation and were therefore in a very immature stage of development. Enzymatic digestion of the testicular tissue resulted in a heterogeneous cell suspension composed of germ cells at early developmental stage and somatic cells (mainly blood cells), but the use of Percoll gradient enriched the spermatogonia cell population. In this sense, the Percoll gradient centrifugation positioned the spermatogonial cells in the middle layer, whereas lipids were in the upper layer and somatic cells pelleted down at the bottom of the tube. Many somatic cells were removed from the spermatogonial cell suspension, but the complete removal could not be achieved. The viability test revealed that 93.5 ± 1.7% of isolated spermatogonia survived the process.

#### mRNA expression

Intra-assay coefficient of variation (CV, %) for each gene (*vasa1*, *vasa2*, *nanos2*, *dnd1)* and tissue (muscle, testis, and spermatogonia fraction) was calculated. The mean % CV for each gene was 0.98% (*vasa1*), 2.99% (*vasa2*), 0.70% (*dnd1*), and 1.80% (*nanos2*).

**Fig. 5:**
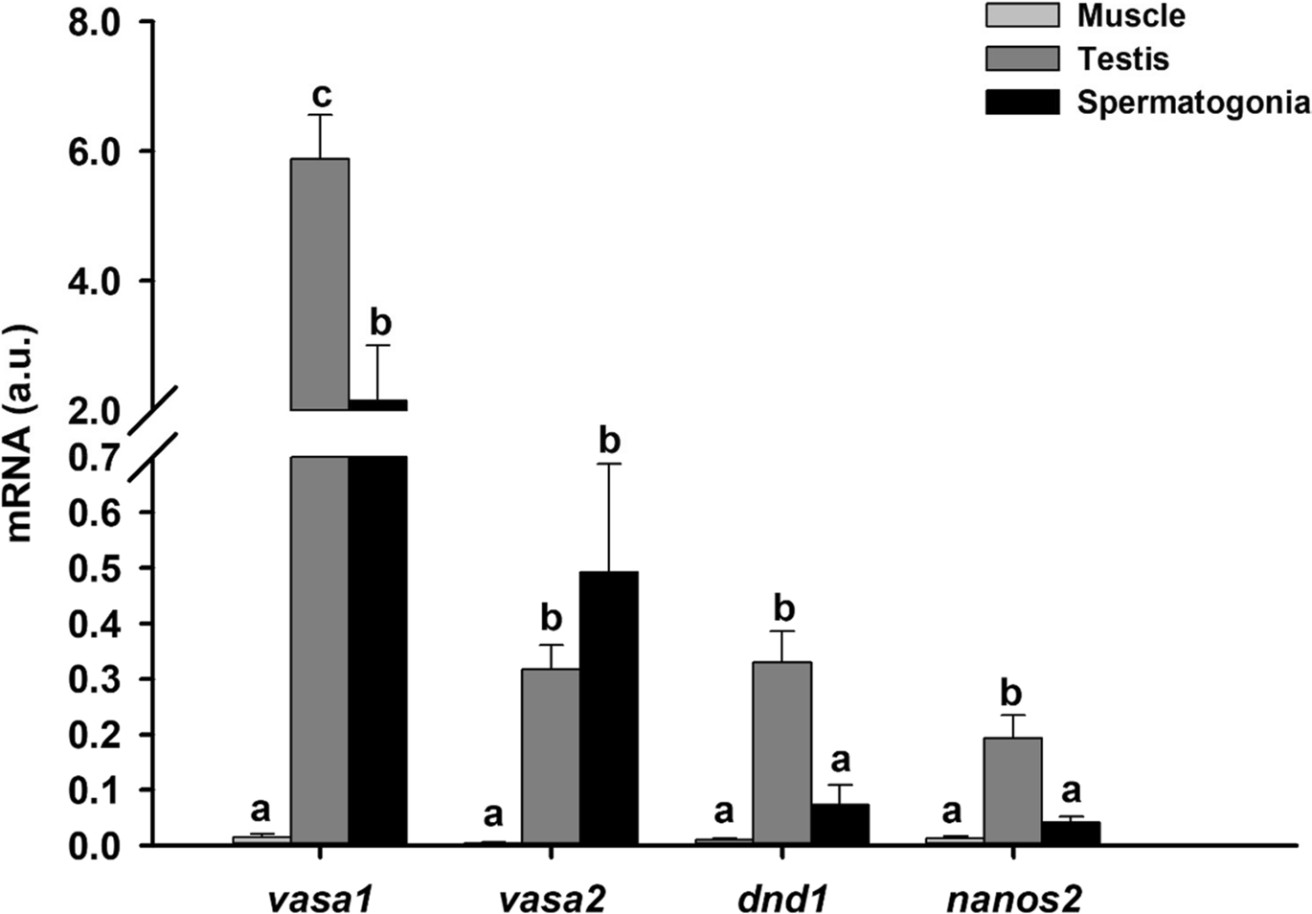
Expression of *vasa1*, *vasa2*, *dnd1*, and *nanos2* mRNA in the muscle, testis, and spermatogonia of immature male European eels, measured by quantitative PCR (qPCR). Data are normalized to eel acidic ribosomal phosphoprotein P0 (ARP), and results are shown as means ± SEM (*n* = 23 for muscle; *n* = 39 for testis; *n* = 13 for spermatogonia). Different letters indicate significant differences (Kruskal–Wallis test; *P* < 0.05)

The expression of *vasa1*, *vasa2*, *dnd1*, and *nanos2* genes was detected in testes, enriched spermatogonia fraction, and muscle, with a significantly lower gene expression in the muscle (Fig. 5). *Vasa1*, *nanos2*, and *dnd1* showed the same expression pattern, with a higher gene expression in the gonad than in the isolated spermatogonia (2.7, 4.5, and 4.8-fold higher, respectively). In contrast, the *vasa2* mRNA transcript did not show significant differences between the gonad and the isolated spermatogonia.

### Gene location of potential biomarkers in testis by FISH

**Fig. 6:**
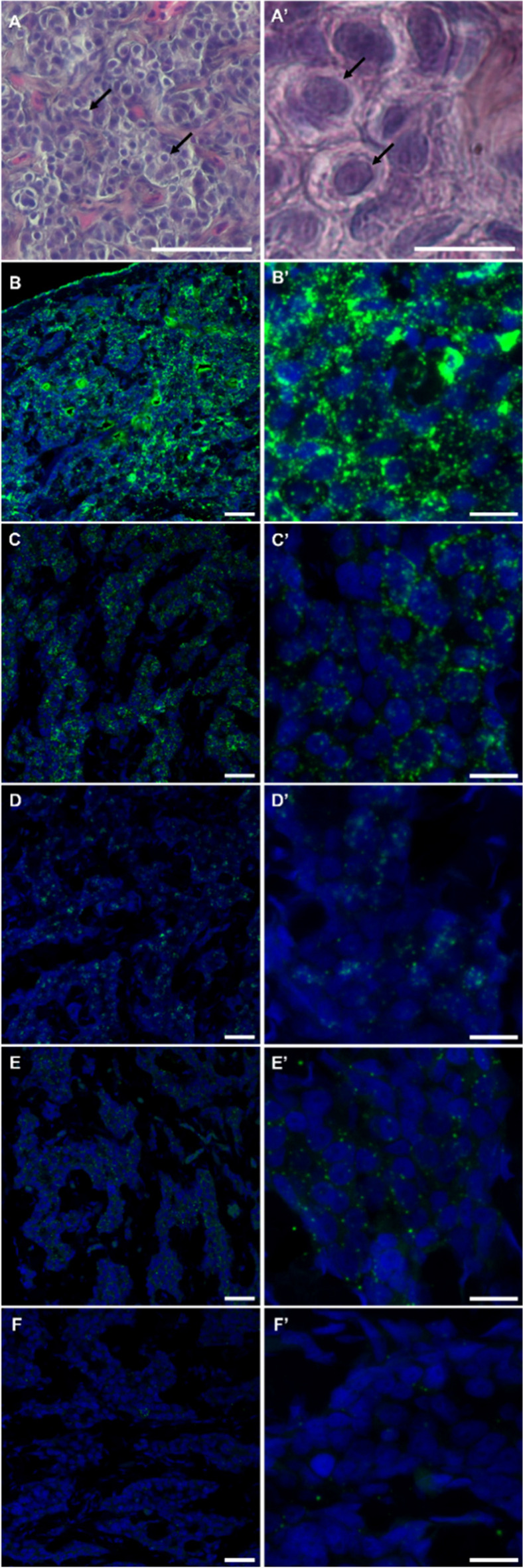
**A**, **A′** Photographs taken with a phase-contrast microscope of histological sections of immature European eel testis, stained with hematoxylin and eosin. Spermatogonia are identified with arrows. Photographs taken with confocal microscope of immature European eel testis for detection of potential spermatogonia markers by FISH, labelled with Opal 250 (dilution 1:750) and DAPI fluorophores. **B**, **B′** *ß-actin* (positive control); **C**, **C′** *vasa1*; **D**, **D′** *vasa2*; **E**, **E′** *dnd1*; **F**, **F′** *nanos2*. Scale bars A, B, C, D, E, and F—25 µm; A′, B′, C′, D′, E′, and F′—10 µm

A preliminary examination of the testis sections was conducted to determine the maturation stage (Fig. 6A, A′), confirming that most contained cells were SPGA. *ß-actin* signal (positive control) was distributed evenly throughout the tissue without cell-specificity (Fig. 6B, B′). Moreover, the signals of *vasa1* (Fig. 6C, C′) and *vasa2* (Fig. 6D, D′) were detected only in SPGA, characterized by their large round shape and pronounced nucleus. Both *vasa1* and *vasa2* signal spots were primarily accumulated in clusters, but *vasa1* signaling spots were more widely distributed than *vasa2* ones. The distribution of *dnd1* (Fig. 6E, E′) and *nanos2* (Fig. 6F, F′) transcripts was also limited to SPGA, but the signal was more dispersed and weaker than those of both *vasa* genes.

**Fig. 7:**
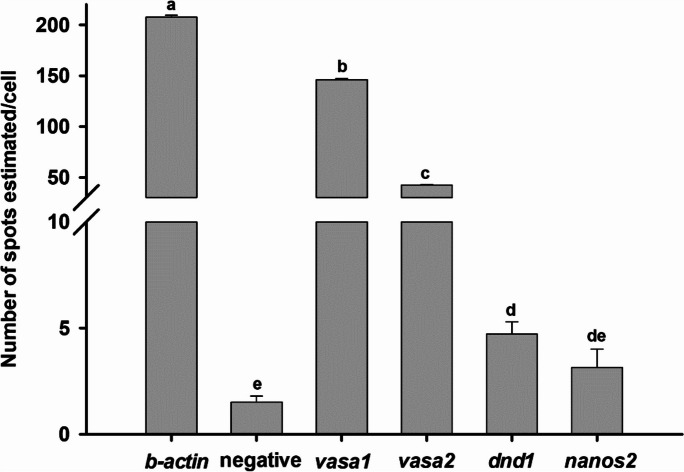
Expression quantification by QuPath software for *β-actin* (positive control), negative control, *vasa1*, *vasa2*, *dnd1*, and *nanos2*, showing the average number of spots estimated per cell. Results are shown as means ± SEM. Different letters indicate significant differences (Kruskal–Wallis test, *P* < 0.05)

The highest number of spots was found for *ß-actin* (positive control, Fig. 6B, B′) which is distributed in the cytoplasm of all cell types, while the negative control did not show fluorescent dots (not shown). The mean number of estimated spots per cell showed significant differences among the potential spermatogonial markers, where the *vasa1*, as well as *vasa2* transcripts displayed a significantly higher number of estimated spots than *dnd1* and *nanos2* (Fig. 7).

### Localization of the potential markers by immunohistochemistry

**Fig. 8:**
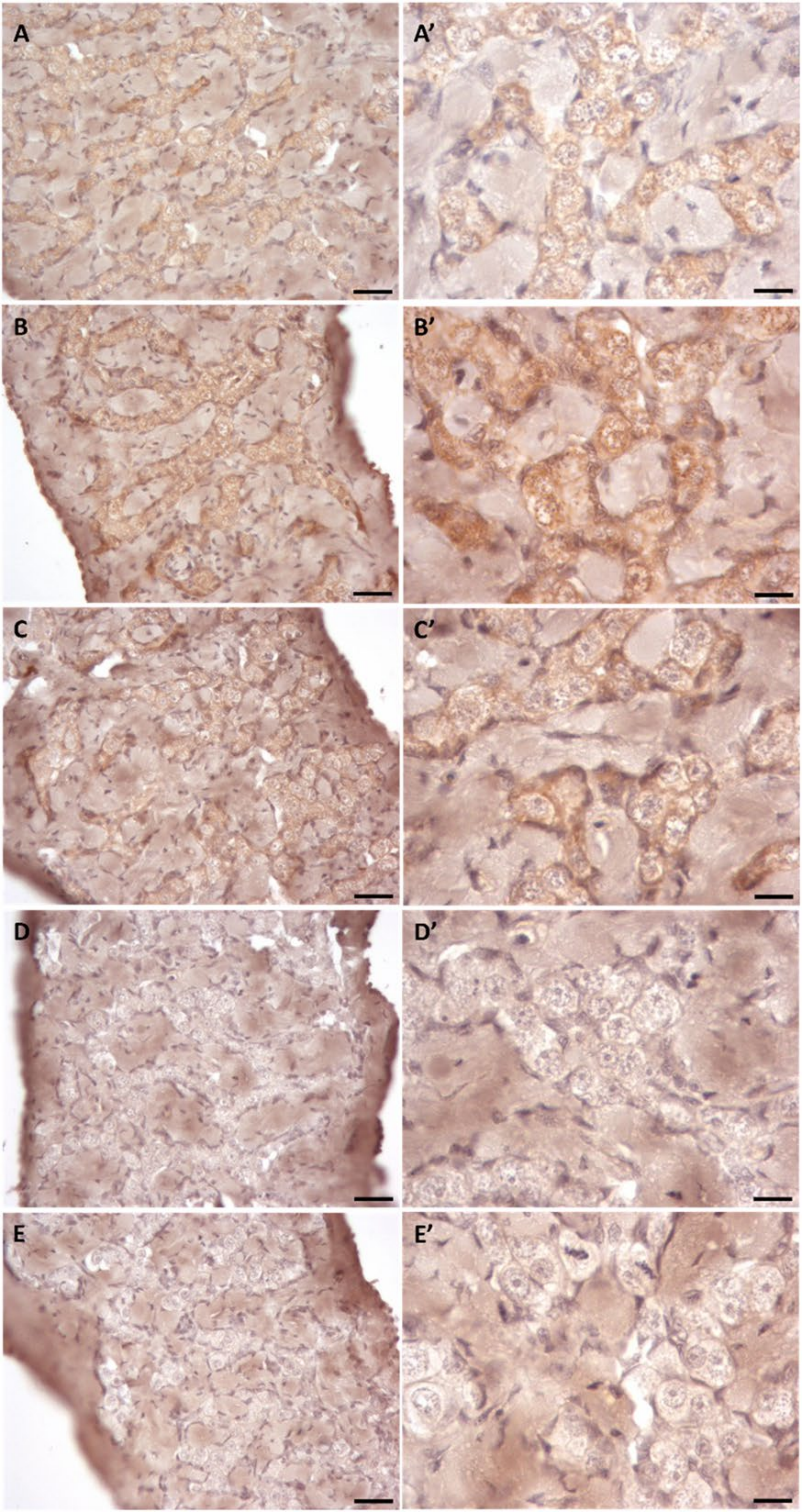
Immunohistochemical detection of Vasa (**A–A′**), Nanos2 (**B–B′**), and Dnd1 (**C–C′**) antigens in the immature testicular tissue of European eel, as well as the secondary antibody control (**D–D′**) and the DAB control (**E–E′**). Each panel under the same letter represents a different magnification. Scale bars A, B, C, D, and E—25 µm; A′, B′, C′, D′, and E′—10 µm

Immunohistochemistry localization of Vasa, Nanos2, and Dnd1 proteins in the immature European eel testicular tissue conducted through the immunoperoxidase visualization method displayed that all three investigated markers were localized specifically in the germline cells (Fig. 8). As the only germline cells in the immature European eel testicular tissue are SPGA, the three markers were localized within these cells. However, the intensity varied significantly; while the signals for Vasa and Nanos2 were strong, the signal for Dnd, even at a 1:10 dilution ratio, was very weak. As the dilution ratio for the anti-vasa antibody was the highest but still resulted in a strong signal, this protein was the most favorable among the tested ones. Even though the antibodies used in this study were developed against mammalian antigens, their labelling pattern was in accordance with FISH results thus further confirming their specificity to European eel antigens as well. Background DAB staining was noticed in the connective tissue of all samples, including the controls; however, this is a regular occurrence in DAB staining and is not representative of an actual localization signal.

**Fig. 9:**
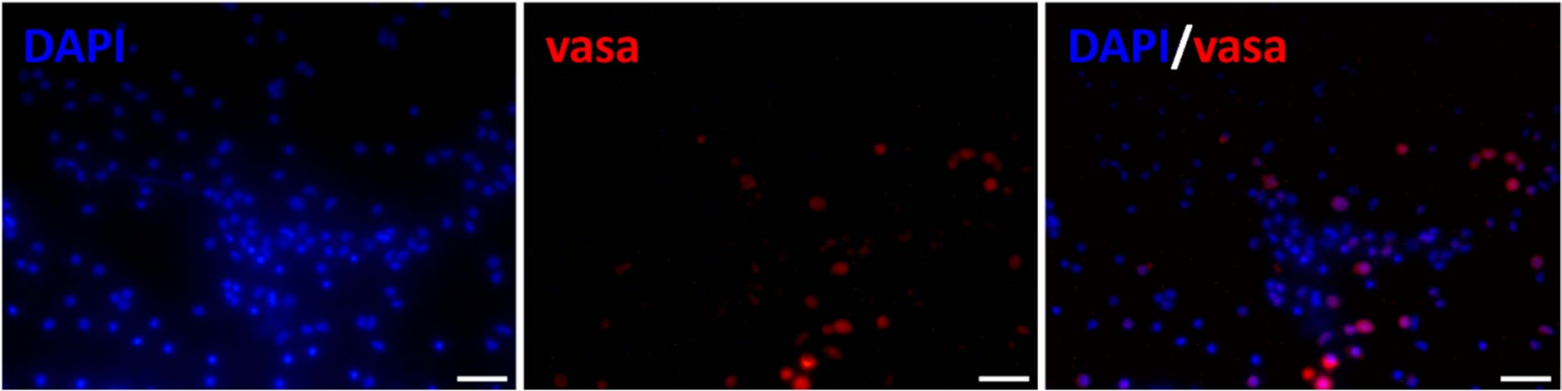
Immunocytochemical detection of the Vasa-positive cells (displaying red fluorescence) in the testicular suspensions of the immature European eel using DAPI staining (blue fluorescence). Scale bars, 25 µm

As Vasa was identified as the most suitable SPGA marker in immature European eel testicular tissues by FISH and immunohistochemistry, we tested the efficiency of this marker to identify isolated SPGA within the testicular cell suspension. By immunocytochemistry, we could observe that the signal was detected only in cells with large round nuclei corresponding to the SPGA (Fig. 9), and not in other somatic cells.

## Discussion

### Duplicated vasa genes in the eel

Few studies reported phylogenetic analyses of potential spermatogonial biomarkers in teleosts, including the DEAD-box helicase family (Vasconcelos et al. 2019; Xu et al. 2005; Ye et al. 2007). To better understand the evolutionary history of the DEAD-box helicase family, we performed phylogenetic analyses on vertebrate amino acid sequences of two members of this family, Vasa and DDX3X. Duplicated *vasa* and *ddx3x* genes have been retrieved from eel genomes. Two *ddx3x* paralogs were also present in other teleost species, while only one *vasa* was retrieved in the other teleost non-elopomorph species. Two events of whole-genome duplication (WGD) (“1R” and “2R”) occurred in ancestral vertebrates (Nakatani et al. 2007), and a third WGD (“3R”) occurred in the teleost linage (Henkel et al. 2012a; Meyer and Van de Peer 2005; Morini et al. 2022). So, two hypotheses could be considered: duplicated eel DDX3X and Vasa may originate either from teleost 3R or from a specific gene duplication that could have occurred in Elopomorphs or Anguillids. In the first hypothesis, one *vasa* paralog gene would have been lost early after the 3R in the teleost lineage.

Phylogenetic analyses were also performed to characterize eel Dnd1 and Nanos2 among vertebrates. Our in silico analysis revealed the presence of a single *dnd1* gene in European and Japanese eels and in other vertebrates. The presence of a single gene in the eels, in teleosts, in non-teleost actinopterygian, and sarcopterygian species reveals no impact of the TWGD on teleost *dnd1* gene number. This suggests that one of the two *dnd1* paralogs would have been lost early after the TWGD in the teleost lineage. The same hypothesis can arise for the *nanos2* evolutive history.

### European eel vasa, dnd1, and nanos2 are expressed in spermatogonial cells

*Vasa* is considered one of the essential markers for identifying germ cells within the animal kingdom (Begum et al. 2022). A species-specific expression pattern of *vasa* mRNA and protein has been observed in PGCs and germ cells of the ovary and testis in diverse fish species (Cao et al. 2012; Duangkaew et al. 2019; Xu et al. 2005; Yuan et al. 2014). In Japanese eel, *vasa* expression occurs mainly in males during testis differentiation but also in the developing ovary of E2-induced feminizing eels (Jeng et al. 2018). However, in the rice field eel, *vasa* was expressed in oocytes at all stages of oogenesis, including degenerating oocytes of ovotestis and in spermatogonia and primary spermatocytes (Ye et al. 2007). This observation highlights the significance of *vasa* and its potential for a better understanding of the reproductive biology in these organisms.

In present study, we characterized two paralogs of *vasa* in the European eel*,* while only one *vasa* gene has been described in vertebrates until now. European eel *vasa1* and *vasa2* showed a higher expression in the testis than in muscle. According to the bibliography, *vasa* expression is mainly restricted to the testis and ovary in adult teleosts, but it can also be expressed in other tissues, although at virtually undetectable levels (Blázquez et al. 2011; Nagasawa et al. 2013). Nevertheless, *vasa1* showed a higher expression in the testis than in spermatogonia while no differences were found in *vasa2*. Moreover, FISH results showed that both *vasa1* and *vasa2* are found in SPGA, while immunohistochemistry and immunocytochemistry confirmed that Vasa protein is also expressed in the SPGA. These results suggested that both *vasa* genes may be involved in the reproductive process (Wang et al. 2022; Xu et al. 2014), and the conservation of duplicated eel *vasa* may reflect evolutionary processes such as neo- or sub-functionalization, as showed in other duplicated genes in eels (Jolly et al. 2016; Nakamura et al. 2017; Maugars and Dufour 2015). Further syntenic studies are needed to clarify the origin of *vasa* genes in teleosts. Together with our qPCR results, both genes could be reliable markers for SPGA in the European eel.

*Nanos* genes are expressed in germ cell lineage in metazoans. Three *nanos* gene subtypes (*nanos1*, *nanos2*, and *nanos3*) have been described in vertebrates. In mice, *nanos2* was mainly expressed in the male germ cells, and knock-out of *nanos2* led to the lack of spermatogonia (Tsuda et al. 2003, 2006). In fish, depending on the species, specifically the expression of *nanos2* and *nanos3* mRNA and protein has been observed in testicular and ovary germ cells (Aoki et al. 2009; Beer and Draper 2013; Bellaiche et al. 2014; Han et al. 2018; Nakamura et al. 2010; Tsuda et al. 2003). In the European eel, our qPCR analyses revealed that *nanos2* was more expressed in the whole testis compared to the enriched spermatogonia fraction, and low signal was found by the FISH technique.

While *nanos2* is recognized as a marker for SPGA in teleosts, the role of *nanos3* as a specific spermatogonia marker remains unclear (Bellaiche et al. 2014; Han et al. 2018; Lacerda et al. 2014). Despite that, the present study showed that the *nanos2* expression in European eel spermatogonia is low. As a result, the characterization of European eel *nanos3* in testis may be helpful to find a potential germ cell marker inside the *nanos* gene family.

Dead end (*dnd*) gene has been identified as a specific spermatogonial marker in some teleosts (Baloch et al. 2019; Duan et al. 2015; Lin et al. 2013; Yang et al. 2015; Yazawa et al. 2013; Yoshizaki et al. 2016) which suggests that the *dnd* gene may play an important role in spermatogenesis. In our study, eel *dnd1* was identified as orthologous to the other teleost *dnd1*, suggesting a similar function than in other species (Booncherd et al. 2024; Wargelius et al. 2016; Zhu et al. 2018). In this sense, a higher *dnd1* expression level has been revealed in the immature testis of the eels than in the isolated spermatogonia. According to our FISH results, *nanos2* and *dnd1* may not be reliable molecular markers for identifying SPGA in the European eel, as they present a weak signal compared to *vasa1* and *vasa2*. Previous studies in mammals have shown that the dissociation of human spermatogonial stem cells from their niche may alter their gene expression profile to a considerable degree, compared with spermatogonia in the in vivo situation (von Kopylow et al. 2010). Considering this, it is difficult to compare the gene expression between whole testis tissue, even it is mostly composed of spermatogonia, and an enriched population of spermatogonia. In the present study, we hypothesize that a higher expression of germ markers (*vasa1*, *nanos2*, and *dnd1*) in the whole testis compared to the enriched spermatogonia fraction is due to the alteration that the dissociation protocol could induce on the spermatogonia expression.

## Conclusions

The expression pattern of spermatogonial markers studied so far appears to be dependent on the species, the sex, and the maturing stage in teleosts. In the European eel, two *vasa* (*vasa1* and *vasa2*), one *nanos2*, and one *dnd1* genes were identified and characterized. All three markers were targeted at the testicular tissue and the enriched spermatogonia fraction. In addition, it was detected some differences in the expression among the markers, concluding that European eel *vasa1* and *vasa2* appeared to be the best early-stage germ cell markers of those tested in immature testes.

## Supplementary Information

Below is the link to the electronic supplementary material.

Supplementary file1 (DOCX 894 KB)

## Data Availability

Not applicable.

## References

[CR1] Abascal F, Zardoya R, Posada D (2005) ProtTest: selection of best-fit models of protein evolution. Bioinformatics 21:2104–2105. 10.1093/bioinformatics/bti263 15647292 10.1093/bioinformatics/bti263

[CR2] Aoki Y, Nakamura S, Ishikawa Y, Tanaka M (2009) Expression and syntenic analyses of four *nanos* genes in medaka. Zool Sci 6:112–118. 10.2108/zsj.26.112 10.2108/zsj.26.112 19341327

[CR3] Asturiano JF (2020) Improvements on the reproductive control of the European eel. In: Yoshida M, Asturiano JF (eds) Reproduction in aquatic animals: from basic biology to aquaculture technology. Springer Singapore, Singapore, p 293–320

[CR4] Baloch AR, Franěk R, Tichopád T, Fučíková M, Rodina M, Pšenička M (2019) Dnd1 knockout in sturgeons by CRISPR/Cas9 generates germ cell free host for surrogate production. Animals 9:74. 10.3390/ani9040174 30999629 10.3390/ani9040174 PMC6523263

[CR5] Bankhead P, Loughrey MB, Fernández JA, Dombrowski Y, McArt DG, Dunne PD, McQuaid S, Gray RT, Murray LJ, Coleman HG, James JA, Salto-Tellez M, Hamilton PW (2017) QuPath: Open source software for digital pathology image analysis. Sci Rep 7:1–7. 10.1038/s41598-017-17204-5 29203879 10.1038/s41598-017-17204-5 PMC5715110

[CR6] Beer RL, Draper BW (2013) *Nanos3* maintains germline stem cells and expression of the conserved germline stem cell gene *nanos2* in the zebrafish ovary. Dev Biol 374:308–318. 10.1016/j.ydbio.2012.12.003 23228893 10.1016/j.ydbio.2012.12.003

[CR7] Begum S, Gnanasree SM, Anusha N, Senthilkumaran B (2022) Germ cell markers in fishes – a review. Aquaculture and Fisheries 7:540–552. 10.1016/j.aaf.2022.03.015

[CR8] Bellaiche J, Lareyre JJ, Cauty C, Yano A, Allemand I, Le Gac F (2014) Spermatogonial stem cell quest: *nanos2*, marker of a subpopulation of undifferentiated A spermatogonia in trout testis. Biol Reprod 79:1–14. 10.1095/biolreprod.113.116392 10.1095/biolreprod.113.116392 24554733

[CR9] Blázquez M, González A, Mylonas CC, Piferrer F (2011) Cloning and sequence analysis of *vasa* homolog in the European sesa bass (*Dicentrarchus labrax*): tissue distribution and mRNA expression levels during early development and sex differentiation. Gen Com Endocrinol 170:322–333. 10.1016/j.ygcen.2010.10.007 10.1016/j.ygcen.2010.10.007 20955711

[CR10] Booncherd K, Sreebun S, Pasomboon P, Boonanuntanasarn S (2024) Effects of CRISPR/Cas9-mediated *dnd1* knockout impairs gonadal development in striped catfish. Animal 18. 10.1016/j.animal.2023.101039 10.1016/j.animal.2023.101039 38103430

[CR11] Bosseboeuf A, Gautier A, Auvray P, Mazan S, Sourdaine P (2014) Characterization of spermatogonial markers in the mature testis of the dogfish (*Scyliorhinus canicula* L.). Reproduction 147:125–139. 10.1530/REP-13-0316 24123129 10.1530/REP-13-0316

[CR12] Burgerhout E, Lokman PM, van den Thillart GEEJM, Dirks RP (2019) The time-keeping hormone melatonin: a possible key cue for puberty in freshwater eels (*Anguilla* spp.). Rev Fish Biol Fisheries 29:1–21. 10.1007/s11160-018-9540-3

[CR13] Cao M, Yang Y, Xu H, Duan J, Cheng N, Wang J, Hu W, Zhao H (2012) Germ cell specific expression of Vasa in rare minnow, *Gobiocypris rarus*. Comp Biochem Physiol A 162:163–170. 10.1016/j.cbpa.2012.02.007 10.1016/j.cbpa.2012.02.007 22357168

[CR14] Castrillon DH, Quade BJ, Wang TY, Quigley C, Crum CP (2000) The human vasa gene is specifically expressed in the germ cell lineage. Proc Natl Acad Sci USA 97(9585):9590. 10.1073/pnas.160274797 10.1073/pnas.160274797 PMC16908 10920202

[CR15] Dekker W (2002) Monitoring of glass eel recruitment. Netherlands: Institute of Fisheries Research, report C007/02-WD

[CR16] Draper BW (2017) Identification of germ-line stem cells in zebrafish. Methods Mol Biol 1463:103–113. 10.1007/978-1-4939-4017-2_8 27734351 10.1007/978-1-4939-4017-2_8

[CR17] Duan J, Feng G, Chang P, Zhang X, Zhou Q, Zhong X, Qi C, Xie S, Zhao H (2015) Germ cell-specific expression of dead end (*dnd*) in rare minnow (*Gobiocypris rarus*). Fish Physiol Biochem 41:561–571. 10.1007/s10695-015-0029-x 25663436 10.1007/s10695-015-0029-x

[CR18] Duangkaew R, Jangprai A, Ichida K, Yoshizaki G, Boonanuntanasarn S (2019) Characterization and expression of *vasa* homolog in the gonads and primordial germ cells of the striped catfish (*Pangasianodon hypophthalmus*). Theriogenology 131:61–71. 10.1016/j.theriogenology.2019.01.022 30947076 10.1016/j.theriogenology.2019.01.022

[CR19] Dufour S, Lopez E, Le Menn F, Le Belle N, Baloche S, Fontaine YA (1988) Stimulation of gonadotropin release and of ovarian development, by the administration of a gonadoliberin agonist and of dopamine antagonists, in female silver eel pretreated with estradiol. Gen Comp Endocrinol 70:20–30. 10.1016/0016-6480(88)90090-1 3286369 10.1016/0016-6480(88)90090-1

[CR20] Gentile L, Casalini A, Emmanuele P, Brusa R, Zaccaroni A, Mordenti O (2022) Gonadal development in European eel populations of North Adriatic lagoons at different silvering stages. Appl Sci 12:2820. 10.3390/app12062820

[CR21] Han K, Chen S, Cai M, Jiang Y, Zhang Z, Wang Y (2018) *Nanos3* not *nanos1* and *nanos2* is a germ cell marker gene in large yellow croaker during embryogenesis. Comp Biochem Physiol A 218:13–22. 10.1016/j.cbpb.2018.01.002 10.1016/j.cbpb.2018.01.002 29331522

[CR22] Hay B, Jan LY, Jan YN (1988) A protein component of *Drosophila* polar granules is encoded by *vasa* and has extensive sequence similarity to ATP-dependent helicases. Cell 55:577–587. 10.1016/0092-8674(88)90216-4 3052853 10.1016/0092-8674(88)90216-4

[CR23] Henkel CV, Burgerhout E, de Wijze DL, Dirks RP, Minegishi Y, Jansen HJ, Spaink HP, Dufour S, Weltzien F-A, Tsukamoto K, van den Thillart GEEJM (2012a) Primitive duplicate Hox clusters in the European eel’s genome. PLoS ONE 7:e32231. 10.1371/journal.pone.0032231 22384188 10.1371/journal.pone.0032231 PMC3286462

[CR24] Henkel CV, Dirks RP, de Wijze DL, Minegishi Y, Aoyama J, Jansen HJ, Turner B, Dufour S, Tsukamoto K, Spaink HP, van den Thillart GE (2012b) First draft genome sequence of the Japanese eel, *Anguilla japonica*. Gene 511:195–201. 10.1371/journal.pone.0032231 23026207 10.1016/j.gene.2012.09.064

[CR25] Howroyd P, Hoyle-Thacker R, Lyght O, Williams D, Kleymenova E (2005) Morphology of the fetal rat testis preserved in different fixatives. Toxicology Pathology 33:300–304. 10.1080/01926230590896145 10.1080/01926230590896145 15902974

[CR26] Jeng SR, Wu GC, Yueh WS, Kuo SF, Dufour S, Chang CF (2018) Gonadal development and expression of sex-specific genes during sex differentiation in the Japanese eel. Gen Comp Endocrinol 257:74–85. 10.1016/j.ygcen.2017.07.031 28826812 10.1016/j.ygcen.2017.07.031

[CR27] Jolly C, Rousseau K, Prézeau L, Vol C, Tomkiewicz J, Dufour S, Pasqualini C (2016) Functional characterisation of eel dopamine D_2_ receptors and involvement in the direct inhibition of pituitary gonadotropins. J Neuroendocrinol 28. 10.1111/jne.12411 10.1111/jne.12411 27453551

[CR28] Kobayashi T, Kajiura-Kobayashi H, Nagahama Y (1998) A novel stage-specific antigen is expressed only in early stages of spermatogonia in Japanese eel, *Anguilla japonica* testis. Mol Reprod Dev 51:355–361. 10.1002/(SICI)1098-2795(199812)51:4%3c355::AID-MRD1%3e3.0.CO;2-G 9820193 10.1002/(SICI)1098-2795(199812)51:4<355::AID-MRD1>3.0.CO;2-G

[CR29] Lacerda SMSN, Costa GMJ, da Silva MdA, Campos-Junior PHA, Segatelli TM, Peixoto MTD, Resende RR, de França LR (2013) Phenotypic characterization and in vitro propagation and transplantation of the Nile tilapia (*Oreochromis niloticus*) spermatogonial stem cells. Gen Comp Endocrinol 192:95–106. 10.1016/j.ygcen.2013.06.013 23792279 10.1016/j.ygcen.2013.06.013

[CR30] Lacerda SMdSN, Costa GMJ, de França LR (2014) Biology and identity of fish spermatogonial stem cell. Gen Comp Endocrinol 207:56–65. 10.1016/j.ygcen.2014.06.018 24967950 10.1016/j.ygcen.2014.06.018

[CR31] Lacerda SMdSN, Aponte PM, Costa GMJ, Campos-Junior PHA, Segatelli TM, da Silva MdA, de França LR (2018) An overview of spermatogonial stem cell physiology, niche and transplantation in fish. Anim Reprod 9:798–808

[CR32] Lasko PF, Ashburner M (1988) The product of the Drosophila gene *vasa* is very similar to eukaryotic initiation factor-4A. Nature 335:611–617. 10.1038/335611a0 3140040 10.1038/335611a0

[CR33] Lin F, Zhao C, Xu S, Ma D, Xiao Z, Xiao Y, Xu C, Liu Q, Li J (2013) Germline-specificand sexually dimorphic expression of a dead end gene homologue in turbot (*Scophthalmus maximus*). Theriogenology 80:665–672. 10.1016/j.theriogenology.2013.06.016 23906483 10.1016/j.theriogenology.2013.06.016

[CR34] Maugars G, Dufour S (2015) Demonstration of the coexistence of duplicated *lh* receptors in teleosts, and their origin in ancestral actinopterygians. PLoS ONE 10. 10.1371/journal.pone.0135184 10.1371/journal.pone.0135184 PMC4536197 26271038

[CR35] Meyer A, Van de Peer Y (2005) From 2R to 3R: evidence for a fish-specific genome duplication (FSGD). BioEssays 27:937–945. 10.1002/bies.20293 10.1002/bies.20293 16108068

[CR36] Mochizuki K, Nishimiya-Fujisawa C, Fujisawa T (2001) Universal occurrence of the *vasa*-related genes among metazoans and their germline expression in Hydra. Dev Genes Evol 211:299–308. 10.1007/s004270100156 11466525 10.1007/s004270100156

[CR37] Morini M, Peñaranda DS, Vílchez MC, Gallego V, Nourizadeh-Lillabadi R, Asturiano JF, Weltzien FA, Pérez L (2015) Transcript levels of the soluble sperm factor protein phospholipase C zeta 1 (PLCζ1) increase through induced spermatogenesis in European eel. Comp Biochem Physiol A 187:168–176. 10.1016/j.cbpa.2015.05.028 10.1016/j.cbpa.2015.05.028 26051612

[CR38] Morini M, Bergqvist CA, Asturiano JF, Larhammar D, Dufour S (2022) Dynamic evolution of transient receptor potential vanilloid (TRPV) ion channel family with numerous gene duplications and losses. Front Endocrinol 13:1013868. 10.3389/fendo.2022.1013868 10.3389/fendo.2022.1013868 PMC9664204 36387917

[CR39] Nagasawa K, Shikina S, Takeuchi Y, Yoshizaki G (2010) Lymphocyte antigen 75 (Ly75/CD205) is a surface marker on mitotic germ cells in rainbow trout. Biol Reprod 83:597–606. 10.1095/biolreprod.109.082081 20554922 10.1095/biolreprod.109.082081

[CR40] Nagasawa K, Miwa M, Yazawa R, Morita T, Takeuchi Y, Yoshizaki G (2012) Characterization of lymphocyte antigen 75 (Ly75/CD205) as a potential cell-surface marker on spermatogonia in Pacific bluefin tuna *Thunnus orientalis*. Fisheries Sci 78:791–800. 10.1007/s12562-012-0501-9

[CR41] Nagasawa K, Fernandes JMO, Yoshizaki G, Miwa M, Babiak I (2013) Identification and migration of primordial germ cells in Atlantic salmon, *Salmo salar*: characterization of *vasa*, *dead end*, and *lymphocyte antigen 75* genes. Mol Reprod Dev 80:118–131. 10.1002/mrd.22142 23239145 10.1002/mrd.22142 PMC3664433

[CR42] Nakamura S, Kobayashi K, Nishimura T, Higashijima S-i, Tanaka M (2010) Identification of germline stem cells in the ovary of the teleost medaka. Science 328:1561–1563. 10.1126/science.1185473 20488987 10.1126/science.1185473

[CR43] Nakamura Y, Yasuike M, Mekuchi M, Iwasaki Y, Ojima N, Fujiwara A, Chow S, Saitoh K (2017) Rhodopsin gene copies in Japanese eel originated in a teleost-specific genome duplication. Zool Lett 3. 10.1186/s40851-017-0079-2 10.1186/s40851-017-0079-2 PMC5645911 29075512

[CR44] Nakatani Y, Takeda H, Kohara Y, Morishita S (2007) Reconstruction of the vertebrate ancestral genome reveals dynamic genome reorganization in early vertebrates. Genome Res 17:1254–1265. 10.1101/gr.6316407 17652425 10.1101/gr.6316407 PMC1950894

[CR45] Okamura A, Yamada Y, Yokouchi K, Horie N, Mikawa N, Utoh T, Tanaka S, Tsukamoto K (2007) A silvering index for the Japanese eel *Anguilla japonica*. Environ Biol Fish 80:77–89. 10.1007/s10641-006-9121-5

[CR46] Ozaki Y, Saito K, Shinya M, Kawasaki T, Sakai N (2011) Evaluation of *Sycp3, Plzf* and *Cyclin B3* expression and suitability as spermatogonia and spermatocyte markers in zebrafish. Gene Expr Patterns 11:309–315. 10.1016/j.gep.2011.03.002 21402175 10.1016/j.gep.2011.03.002

[CR47] Palstra AP, Guerrero MA, de Laak G, Breteler JPGK, van den Thillart GEEJM (2011) Temporal progression in migratory status and sexual maturation in European silver eels during downstream migration. Fish Physiol Biochem 37:285–296. 10.1007/s10695-011-9496-x 21556699 10.1007/s10695-011-9496-x PMC3107437

[CR48] Peñaranda DS, Pérez L, Gallego V, Barrera R, Jover M, Asturiano JF (2010) European eel sperm diluent for short-term storage. Reprod Domest Anim 45:407–415. 10.1111/j.1439-0531.2008.01206.x 18954399 10.1111/j.1439-0531.2008.01206.x

[CR49] Peñaranda DS, Gallego V, Rozenfeld C, Herranz-Jusdado JG, Pérez L, Gómez A, Giménez I, Asturiano JF (2018) Using specific recombinant gonadotropins to induce spermatogenesis and spermiation in the European eel (*Anguilla anguilla*). Theriogenology 107:6–20. 10.1016/j.theriogenology.2017.11.002 29120707 10.1016/j.theriogenology.2017.11.002

[CR50] Robles V, Riesco MF, Psenicka M, Saito T, Valcarce DG, Cabrita E, Herráez P (2017) Biology of teleost primordial germ cells (PGCs) and spermatogonia: biotechnological applications. Aquaculture 472:4–20. 10.1016/j.aquaculture.2016.03.004

[CR51] Sánchez-Sánchez AV, Camp E, García-España A, Leal-Tassias A, Mullor JL (2010) Medaka Oct4 is expressed during early embryo development, and in primordial germ cells and adult gonads. Dev Dyn 239:672–679. 10.1002/dvdy.22198 20034054 10.1002/dvdy.22198

[CR52] Schmidt J (1923) Breeding places and migration of the eel. Nature 111:51–54. 10.1038/111051a0

[CR53] Schulz RW, de França LR, Lareyre J-J, Le Gac F, Chiarini-García H, Nobrega RH, Miura T (2010) Spermatogenesis in fish. 165:390 411 10.1016/j.ygcen.2009.02.013 10.1016/j.ygcen.2009.02.013 19348807

[CR54] Schüpbach T, Wieschaus E (1986) Germline autonomy of maternal effect mutations altering the embryonic body pattern of *Drosophila*. Dev Biol 113:443–448. 10.1016/0012-1606(86)90179-x 3081391 10.1016/0012-1606(86)90179-x

[CR55] Sievers F, Wilm A, Dineen D, Gibson TJ, Karplus K, Li W, Lopez R, Mc William H, Remmert M, Söding J, Thompson JD, Higgins DG (2011) Fast, scalable generation of high-quality protein multiple sequence alignments using Clustal Omega. Mol Syst Biol 7:539. 10.1038/msb.2011.75 21988835 10.1038/msb.2011.75 PMC3261699

[CR56] Stamatakis A, Ott M (2008) Efficient computation of the phylogenetic likelihood function on multi-gene alignments and multi-core architectures. Philos Trans R Soc B 363:3977–3984. 10.1098/rstb.2008.0163 10.1098/rstb.2008.0163 PMC2607410 18852107

[CR57] Tsuda M, Sasaoka Y, Kiso M, Abe K, Haraguchi S, Kobayashi S, Saga Y (2003) Conserved role of NANOS proteins in germ cell development. Science 301:1239–1241. 10.1126/science.1085222 12947200 10.1126/science.1085222

[CR58] Tsuda M, Kiso M, Saga Y (2006) Implication of nanos2-3’UTR in the expression and function of *nanos2*. Mech Dev 123:440–449. 10.1016/j.mod.2006.04.002 16806845 10.1016/j.mod.2006.04.002

[CR59] van Ginneken VJ, Maes GE (2005) The European eel (*Anguilla anguilla*, Linnaeus), its lifecycle, evolution and reproduction: a literature review. Rev Fish Biol Fisher 15:367–398. 10.1007/s11160-006-0005-8

[CR60] Vasconcelos ACN, Streit DP, Octavera A, Miwa M, Kabeya N, Garcia RRF, Rotili DA, Yoshizaki G (2019) Isolation and characterization of a germ cell marker in teleost fish *Colossoma macropomum*. Gene 683:54–60. 10.1016/j.gene.2018.10.027 30316926 10.1016/j.gene.2018.10.027

[CR61] von Kopylow K, Kirchhoff C, Jezek D, Schulze W, Feig C, Primig M, Steinkraus V, Spiess A-N (2010) Screening for biomarkers of spermatogonia within the human testis: a whole genome approach. Hum Reprod 25:1104–1112. 10.1093/humrep/deq053 20208059 10.1093/humrep/deq053

[CR62] Wang M, Ding H, Wu S, Wang M, Wei C, Wang B, Bao Z, Hu J (2022) *Vasa* is a potential germ cell marker in leopard coral grouper (*Plectropomus leopardus*). Genes 13. 10.3390/genes13061077 10.3390/genes13061077 PMC9222667 35741839

[CR63] Wargelius A, Leininger S, Skaftnesmo KO, Kleppe L, Andersson E, Taranger GL, Schulz R, Edvardsen RB (2016) *Dnd* knockout ablates germ cells and demonstrates germ cell independent sex differentiation in Atlantic salmon. Sci Rep 6. 10.1038/srep21284 10.1038/srep21284 PMC4758030 26888627

[CR64] Weidinger G, Stebler J, Slanchev K, Dumstrei K, Wise C, Lovell-Badge R, Thisse C, Thisse B, Raz E (2003) Dead end, a novel vertebrate germ plasm component, is required for zebrafish primordial germ cell migration and survival. Curr Biol 13:1429–1434. 10.1016/s0960-9822(03)00537-2 12932328 10.1016/s0960-9822(03)00537-2

[CR65] Xu H, Gui J, Hong Y (2005) Differential expression of vasa RNA and protein during spermatogenesis and oogenesis in the gibel carp (*Carassius auratus gibelio*), a bisexually and gynogenetically reproducing vertebrate. Dev Dyn 233:872–882. 10.1002/dvdy.20410 15880437 10.1002/dvdy.20410

[CR66] Xu H, Lim M, Dwarakanath M, Hong Y (2014) *Vasa* identifies germ cells and critical stages of oogenesis in the Asian seabass. Int J Biol Sci 10:225–235. 10.7150/ijbs.6797 24550690 10.7150/ijbs.6797 PMC3927134

[CR67] Yang X, Yue H, Ye H, Li C, Wei Q (2015) Identification of a germ cell marker gene, the dead end homologue, in Chinese sturgeon *Acipenser sinensis*. Gene 558:118–125. 10.1016/j.gene.2014.12.059 25550043 10.1016/j.gene.2014.12.059

[CR68] Yano A, Von Schalburg K, Cooper G, Koop BF, Yoshizaki G (2009) Identification of a molecular marker for type A spermatogonia by microarray analysis using gonadal cells from *pvasa-GFP* transgenic rainbow trout (*Oncorhynchus mykiss*). Mol Reprod Dev 76:246–254. 10.1002/mrd.20947 18646050 10.1002/mrd.20947

[CR69] Yazawa R, Takeuchi Y, Morita T, Ishida M, Yoshizaki G (2013) The Pacific bluefin tuna (*Thunnus orientalis*) dead end gene is suitable as a specific molecular marker of type A spermatogonia. Mol Reprod Dev 80:871–880. 10.1002/mrd.22224 23913406 10.1002/mrd.22224

[CR70] Ye D, Lv D, Song P, Peng M, Chen Y, Guo M, Yang Q, Hu Y (2007) Cloning and characterization of a rice field eel *vasa-*like gene cDNA and its expression in gonads during natural sex transformation. Biochem Genet 45:211–224. 10.1007/s10528-006-9066-6 17318374 10.1007/s10528-006-9066-6

[CR71] Yoshizaki G, Takashiba K, Shimamori S, Fujinuma K, Shikina S, Okutsu T, Kume S, Hayashi M (2016) Production of germ cell-deficient salmonids by dead end gene knockdown, and their use as recipients for germ cell transplantation. Mol Reprod Dev 83:298–311. 10.1002/mrd.22625 26860442 10.1002/mrd.22625

[CR72] Yuan Y, Li M, Hong Y (2014) Light and electron microscopic analyses of Vasa expression in adult germ cells of the fish medaka. Gene 545:15–22. 10.1016/j.gene.2014.05.017 24814190 10.1016/j.gene.2014.05.017

[CR73] Zhu T, Gui L, Zhu Y, Li M (2018) *Dnd* is required for primordial germ cell specification in *Oryzias celebensis*. Gene 679:36–43. 10.1016/j.gene.2018.08.068 30171940 10.1016/j.gene.2018.08.068

