# Supplementary figures and images for: Characterization of potential spermatogonia biomarker genes in the European eel (Anguilla anguilla)
Source: Fish Physiol Biochem. 2024 Apr 19;50(5):2099–115. doi: 10.1007/s10695-024-01338-1 (PMC11576858; doi:10.1007/s10695-024-01338-1)

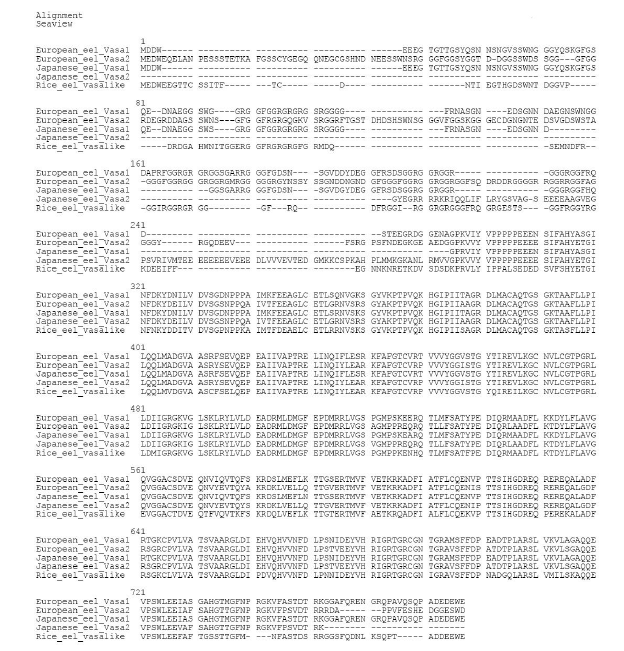


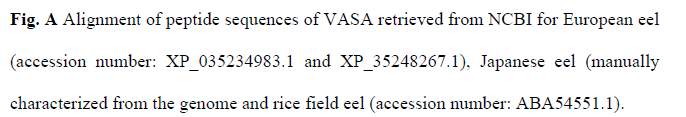


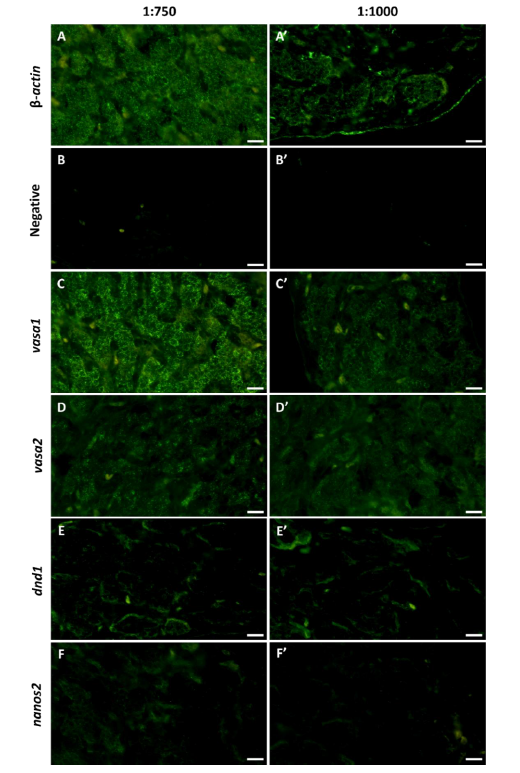


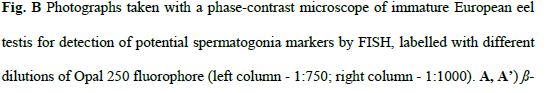

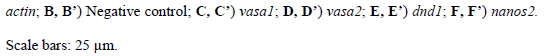


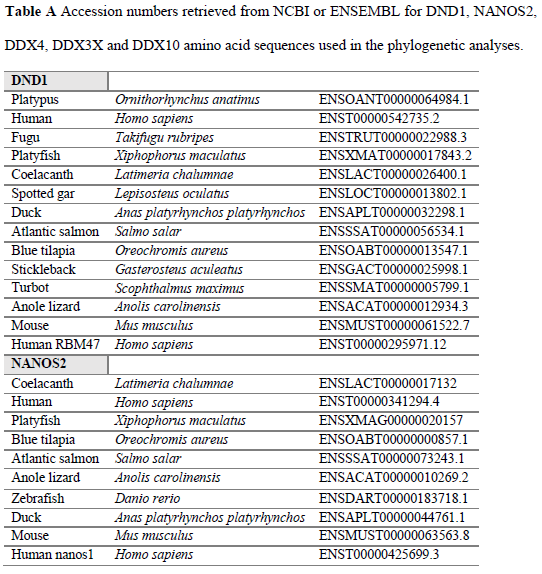


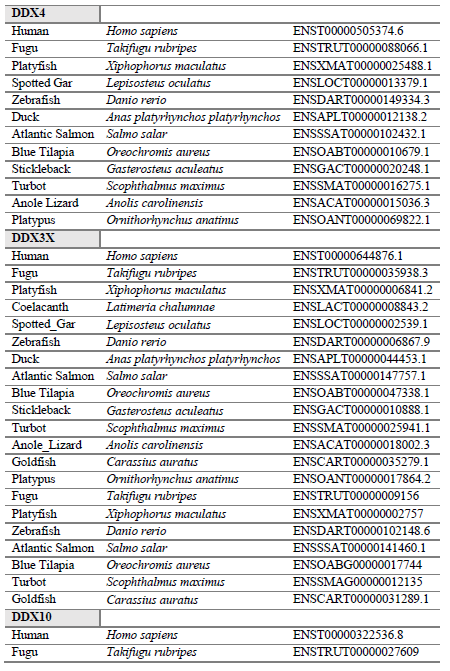

Supplement: Supplementary file 1 — Supplementary file1 (DOCX 894 KB) [file 10695_2024_1338_MOESM1_ESM.docx]
